# Supplementary material for: Mosquitoes (Diptera: Culicidae) in the Dark—Highlighting the Importance of Genetically Identifying Mosquito Populations in Subterranean Environments of Central Europe
Source: Pathogens. 2021 Aug 26;10(9):1090. doi: 10.3390/pathogens10091090 (PMC8467396; doi:10.3390/pathogens10091090)
Supplement: Supplementary file 1 [file pathogens-10-01090-s001.zip › Table_S1.pdf]

Table S1; Mosquitoes sampled in subterranean sites in Luxembourg (T=transition zone, D=dark zone, E=entrance)

ID	taxon	gender	sampling date	sampling site	artificial / natural	cave region
131113 -119	*Cx. pipiens f. pipiens*	f	13.11.2013	Dolomitgrouf Kelsbaach, Machtum	artificial	T
150404 -46	*Cx.torrentium*	f	04.04.2015	Keltenhiel, Müllerthal	natural	T
150404 -47	*Cx. pipiens f. pipiens*	f	04.04.2015	Keltenhiel, Müllerthal	natural	T
080504 -31	*Cx. pipiens f. pipiens*	f	04.05.2008	Grotte de la Vierge, Müllerthal	natural	D
070817 -233	*Cx. pipiens f. pipiens*	f	17.08.2007	Schlöff, Mersch	artificial	T
080315 -249	*Cx. torrentium*	f	15.03.2008	Schlöff, Mersch	artificial	T
081226 -76	*Cx. pipiens f. pipiens*	f	26.12.2008	Minière Laangebierg Diddeleng, Dudelange	artificial	T
081226 -85	*Cx. torrentium*	f	26.12.2008	Minière Laangebierg Diddeleng, Dudelange	artificial	T
090820 -113	*Cx. torrentium*	f	20.08.2009	Fort Berlaimont, Luxembourg	artificial	T
090820 -138	*Cx.* torrentium	f	20.08.2009	Fort Berlaimont, Luxembourg	artificial	T
090820 -142	*Cx. torrentium*	f	20.08.2009	Fort Berlaimont, Luxembourg	artificial	D
111031 -407	*Cx. pipiens f. pipiens*	f	31.10.2011	Fort Vauban, Luxembourg	artificial	T
111031 -406	*Cx. torrentium*	f	31.10.2011	Fort Vauban, Luxembourg	artificial	T
111031 -411	*Cx. pipiens f. pipiens*	f	31.10.2011	Fort Vauban, Luxembourg	artificial	D
111031 -414	*Cx. pipiens f. pipiens*	f	31.10.2011	Fort Vauban, Luxembourg	artificial	D
111031 -416	*Cx. pipiens f. pipiens*	f	31.10.2011	Fort Vauban, Luxembourg	artificial	D
111031 -450	*Cx. pipiens f. pipiens*	f	31.10.2011	Fort Royal, Luxembourg	artificial	D
111031 -453	*Cx. pipiens f. pipiens*	f	31.10.2011	Fort Royal, Luxembourg	artificial	D
111031 -457	Cx. *pipiens* f. pipiens	f	31.10.2001	Fort Royal, Luxembourg	artificial	D
120428 -146	*Cx. pipiens f. molestus*	f	28.04.2012	Fort Royal, Luxembourg	artificial	D
120428 -153	*Cx. pipiens f. pipiens*	f	28.04.2012	Fort Royal, Luxembourg	artificial	T
130107 -11	*Cx. pipiens f. pipiens*	f	07.01.2013	Grassebiergtunnel, Bech	artificial	T
130107 -19	*Cx. pipiens f. pipiens*	f	07.01.2013	Grassebiergtunnel, Bech	artificial	T
130107 -22	*Cx. pipiens f. pipiens*	f	07.01.2013	Grassebiergtunnel, Bech,	artificial	T
130107 -48	*Cx. pipiens f. pipiens*	f	07.01.2013	Grassebiergtunnel, Bech	artificial	T
130107 -51	*Cx. pipiens f. pipiens*	f	07.01.2013	Grassebiergtunnel, Bech	artificial	T
130816 -119_a	Cx. *pipiens f. pipiens*	f	16.08.2013	Fort Rubamprez, Luxembourg	artificial	E
130816 -119_b	*Cx. pipiens* f. pipiens	f	16.08.2013	Fort Rubamprez, Luxembourg	artificial	E
130816 -125_a	*Cx. pipiens f. pipiens*	f	16.08.2013	Fort Rubamprez, Luxembourg	artificial	T

130816-125_b	*Cx. pipiens f. pipiens*	f	16.08.2013	Fort Rubamprez, Luxembourg	artificial	T
130816-125_c	*Cx. pipiens f. pipiens*	f	16.08.2013	Fort Rubamprez, Luxembourg	artificial	T
130816-135_a	*Cx. torrentium*	f	16.08.2013	Fort Rubamprez, Luxembourg	artificial	T
130510-98	*Cx. pipiens f. pipiens*	f	10.05.2013	Verbindungsgang Rahnplateau – Grundschleuse, Luxembourg	artificial	D
131210-12	*Cx. pipiens f. pipiens*	f	10.12.2013	Fort du Moulin, Luxembourg	artificial	T
131210-13	*Cx. pipiens f. pipiens*	f	10.12.2013	Fort du Moulin, Luxembourg	artificial	T
131210-14	*Cx. pipiens f. pipiens*	f	10.12.2013	Fort du Moulin, Luxembourg	artificial	T
131210-15	*Cx. pipiens f. pipiens*	f	10.12.2013	Fort du Moulin, Luxembourg	artificial	T
131210-16	*Cx. pipiens f. pipiens*	f	10.12.2013	Fort du Moulin, Luxembourg	artificial	T
131114-04	*Cx. pipiens f. pipiens*	f	14.11.2013	Keller unter Burgmauer, Echternach	artificial	T
131114-05	*Cx. pipiens f. pipiens*	f	14.11.2013	Keller unter Burgmauer, Echternach	artificial	T
141117-16	*Cx. pipiens f. pipiens*	f	17.11.2014	Keller Assa, Luxembourg	artificial	T
141211-06	*Cx. pipiens f. pipiens*	f	11.12.2014	Höhle 2, Luxemburg	natural	T
141211-07	*Cx. pipiens f. pipiens*	f	11.12.2014	Höhle 2, Luxemburg	natural	T
141211-09	*Cx. pipiens f. pipiens*	f	11.12.2014	Höhle 2, Luxemburg	natural	T
141211-10	*Cx. pipiens f. pipiens*	f	11.12.2014	Höhle 2, Luxemburg	natural	T
141211-19	*Cx. pipiens f. pipiens*	f	11.12.2014	Höhle 3, Luxemburg	natural	T
150417-70	*Cx. pipiens f. pipiens*	f	17.04.2015	Fort Lambert, Luxembourg	artificial	D
150417-96	*Cx. pipiens f. pipiens*	f	17.04.2015	Fort Lambert, Luxembourg	artificial	D
150417-33	*Cx. pipiens f. pipiens*	f	17.042015	Fort Lambert, Luxembourg	artificial	T
150417-34	*Cx. pipiens f. pipiens*	f	17.042015	Fort Lambert, Luxembourg	artificial	T
150417-69	*Cx. pipiens f. pipiens*	f	17.042015	Fort Lambert, Luxembourg	artificial	D
130926-24	*Cx. torrentium*	f	26.09.2013	Gipsminn bei Girsterklaus,	artificial	T
130816-45	*Cx. torrentium*	f	16.08.2013	Bahntunnel, Junglinster	artificial	T
130816-47	*Cx. torrentium*	f	16.08.2013	Bahntunnel, Junglinster	artificial	T
130816-48	*Cx. torrentium*	f	16.08.2013	Bahntunnel, Junglinster	artificial	T
130817-42	*Cx. torrentium*	f	17.08.2013	Bahntunnel, Junglinster	artificial	T
130817-65	*Cx. torrentium*	f	17.08.2013	Bahntunnel, Junglinster	artificial	T
131112-06	*Cx. pipiens f. pipiens*	f	12.11.2013	Galerie de Bockholtzermillen, Goesdorf	artificial	T
131112-07	*Cx. pipiens f. pipiens*	f	12.11.2013	Galerie de Bockholtzermillen, Goesdorf	artificial	T
131112-08	*Cx. pipiens f. pipiens*	f	12.11.2013	Galerie de Bockholtzermillen, Goesdorf	artificial	T
131112-09	*Cx. pipiens f. pipiens*	f	12.11.2013	Galerie de Bockholtzermillen, Goesdorf	artificial	T

121202 -119	*Cx. torrentium*	f	02.12.2012	Antimonminn, Goesdorf	artificial	T
121202 -125_a	*Cx. pipiens f. pipiens*	f	02.12.2012	Antimonminn, Goesdorf	artificial	T
121202 -138	*Cx. torrentium*	f	02.12.2012	Antimonminn, Goesdorf	artificial	D
121202 -145	*Cx. torrentium*	f	02.12.2012	Antimonminn, Goesdorf	artificial	D
100327 -253	*Cx. torrentium*	f	27.03.2010	Schiefergrouf vu Pärel, Perlé	artificial	T
100327 -254	*Cx. torrentium*	f	27.03.2010	Schiefergrouf vu Pärel, Perlé	artificial	T
100327 -255	*Cx. torrentium*	f	27.03.2010	Schiefergrouf vu Pärel, Perlé	artificial	T
100327 -265	*Cx. pipiens f. pipiens*	f	27.03.2010	Schiefergrouf vu Pärel, Perlé	artificial	D
101106 -177	*Cx. torrentium*	f	06.11.2010	Minière Weltschegrond II, Rumelange	artificial	E
101106 -183	*Cx. pipiens f. pipiens*	f	06.11.2010	Minière Weltschegrond II, Rumelange	artificial	T
101231 -19	*Cx. torrentium*	f	31.12.2010	Minière Weltschegrond II, Rumelange	artificial	T
101231 -26	*Cx. pipiens f. pipiens*	f	31.12.2010	Minière Weltschegrond II, Rumelange	artificial	T
080314 -18	*Cx. pipiens f. pipiens*	f	14.03.2008	SME-Tunnel, Rumelange	artificial	T
140328 -118	*Cx. pipiens f. pipiens*	f	28.03.2014	SME-Tunnel, Rumelange	artificial	T
101107 -202	*Cx. torrentium*	f	07.11.2010	Minière Laange Gronn XII, Rumelange	artificial	T
101107 -208	*Cx. torrentium*	f	07.11.2010	Minière Laange Gronn XII, Rumelange	artificial	T
101231 -46	*Cx. torrentium*	f	31.12.2010	Minière Laange Gronn XII, Rumelange	artificial	D
140417 -06	*Cx. pipiens f. pipiens*	f	17.04.2014	Minière Reschelerkopp, Rumelange	artificial	E
141227 -14	*Cx. torrentium*	f	27.12.2014	Minière Reschelerkopp, Rumelange	artificial	T
141227 -15	*Cx. pipiens f. pipiens*	f	27.12.2014	Minière Reschelerkopp, Rumelange	artificial	T
141227 -18	*Cx. pipiens f. pipiens*	f	27.14.2014	Minière Reschelerkopp, Rumelange	artificial	T
141227 -27	*Cx. pipiens f. pipiens*	f	27.12.2014	Minière Reschelerkopp, Rumelange	artificial	D
141230 -20	*Cx. pipiens f. pipiens*	f	30.12.2014	Minière Laange Gronn III, Rumelange	artificial	T
141230 -77	*Cx. pipiens f. pipiens*	f	30.12.2014	Minière Laange Gronn III, Rumelange	artificial	D
141230 -78	*Cx. pipiens f. pipiens*	f	30.12.2014	Minière Laange Gronn III, Rumelange	artificial	D
140327 -09	*Cx. torrentium*	f	37.03.2014	Schifergrouf vu Schläif II, Niederwampach	artifical	T
140327 -35	*Cx. pipiens f. pipiens*	f	27.03.2014	Schifergrouf vu Schläif II, Niederwampach	artificial	T
140327 -121	*Cx. torrentium*	f	27.03.2014	Tussen-Tunnel I, Niederwampach	artificial	T
140327 -135	*Cx. pipiens f. pipiens*	f	27.03.2014	Tussen-Tunnel I, Niederwampach	artificial	T
131214 -41	*Cx. pipiens f. pipiens*	f	14.12.2013	Tunnel Huldange, Weiswampach	artificial	T
131214 -42	*Cx. pipiens f. pipiens*	f	14.12.2013	Tunnel Huldange, Weiswampach	artificial	T
131214 -44	*Cx. pipiens f. pipiens*	f	14.12.2013	Tunnel Huldange, Weiswampach	artificial	D

131214-45	*Cx. torrentium*	f	14.12.2013	Tunnel Huldange, Weiswampach	artificial	D
131214-46	*Cx. torrentium*	f	14.12.2013	Tunnel Huldange, Weiswampach	artificial	D
100206-358	*Cx. pipiens f. pipiens*	f	06.02.2010	Kofferminn Stolzebuerg, Stolzembourg	artificial	D
100206-360	*Cx. torrentium*	f	06.02.2010	Kofferminn Stolzebuerg, Stolzembourg	artificial	D
100206-257_a	*Cx. torrentium*	f	06.02.2010	Kofferminn Stolzebuerg II, Stolzembourg	artificial	T
100206-292	Cx. *torrentium*	f	06.02.2010	Tunnel vun Habscht, Hobscheid	artificial	D
110122-149a	*Cx. pipiens f. pipiens*	f	22.01.2011	Gipsminn, Bettendorf	artificial	T
110122-157	*Cx. pipiens f. pipiens*	f	22.01.2011	Gipsminn, Bettendorf	artificial	T
110122-167	*Cx. pipiens f. pipiens*	f	22.01.2001	Gipsminn, Bettendorf	artificial	T
110122-173	*Cx. torrentium*	f	22.01.2011	Gipsminn, Bettendorf	artificial	T
110909-620	Cx. *torrentium*	f	09.09.2011	Minn vun Asselbuer, Asselborn	artificial	T
111227-03	*Cx. pipiens f. pipiens*	f	27.12.2011	Minn vun Asselbuer, Asselborn	artificial	T
111030-408	*Cx. pipiens f. pipiens*	f	30.10.2011	Minière Doihl, Rodange	artificial	T
111030-422	*Cx. pipiens f. pipiens*	f	30.12.2001	Minière Doihl, Rodange	artificial	T
140328-18	*Cx. pipiens f. pipiens*	f	28.03.2014	Minière de la Crosnière, Lasauvage	artificial	T
140328-19	*Cx. pipiens f. pipiens*	f	28.03.2014	Minière de la Crosnière, Lasauvage	artificial	T
140328-27	*Cx. pipiens f. pipiens*	f	28.03.2014	Minière de la Crosnière, Lasauvage	artificial	T
140328-34	*Cx. pipiens f. pipiens*	f	28.03.2014	Minière de la Crosnière, Lasauvage	artificial	T
140329-105	*Cx. pipiens f. pipiens*	f	29.03.2014	Minière Saintignon, Lasauvage	artificial	T
140329-109	*Cx. pipiens* f. pipiens	f	29.03.2014	Minière Saintignon, Lasauvage	artificial	T
140329-110	Cx. *pipiens f. pipiens*	f	29.03.2014	Minière Saintignon, Lasauvage	artificial	T
140329-113	*Cx. pipiens f. pipiens*	f	29.03.2014	Minière Saintignon, Lasauvage	artificial	T
140329-11	*Cx. pipiens f. pipiens*	f	29.03.2014	Tunnel Lasauvage, Lasauvage	artificial	T
140329-13	*Cx. pipiens f. pipiens*	f	29.03.2014	Tunnel Lasauvage, Lasauvage	artificial	T
140329-42	*Cx. pipiens f. pipiens*	f	29.03.2014	Pulverkammer, Lasauvage	artificial	E
111228-55	*Cx. pipiens f. pipiens*	f	28.12.2011	Wichtelcherslay, Hunnebur	natural	D
120318-40	*Cx. pipiens f. pipiens*	f	18.03.2012	Wichtelcherslay, Hunnebur	natural	D
120730-183	*Cx. pipiens f. pipiens*	f	30.07.2012	Lower dolomite mine, Wasserbillig	artificial	E
120730-199	*Cx. pipiens f. pipiens*	f	30.07.2012	Lower dolomite mine, Wasserbillig	artificial	T
131113-100	*Cx. pipiens f. pipiens*	f	13.11.2013	Upper dolomite mine, Wasserbillig	artificial	T
131113-101	*Cx. pipiens f. pipiens*	f	13.11.2013	Upper dolomite mine, Wasserbillig	artificial	T
131113-102	*Cx. pipiens f. pipiens*	f	13.11.2013	Upper dolomite mine, Wasserbillig	artificial	T

131212-131	*Cx. torrentium*	f	12.12.2013	Déiwepötz, Consdorf	natural	D
131212-134	*Cx. pipiens f. pipiens*	f	12.12.2013	Déiwepötz, Consdorf	natural	D
131212-123	*Cx. pipiens f. pipiens*	f	12.12.2013	Déiwepötz, Consdorf	natural	D
130309-144	*Cx. pipiens f. molestus*	f	09.03.2013	Trou du Bivouak, Consdorf	natural	T
131212-73	*Cx. pipiens f. pipiens*	f	12.12.2013	Trou du Bivouak, Consdorf	natural	T
131212-74	*Cx. pipiens f. pipiens*	f	12.12.2013	Trou du Bivouak, Consdorf	natural	T
130816-98	Cx. *pipiens f. pipiens*	f	16.08.2013	Kuelscheier, Consdorf	natural	T
131212-148	*Cx. pipiens f. pipiens*	f	12.12.2013	Kuelscheier, Consdorf	natural	T
131212-153	*Cx. pipiens f. pipiens*	f	12.12.2013	Kuelscheier, Consdorf	natural	D
130816-108	*Cx. pipiens f. pipiens*	f	16.08.2013	Trou Hélène, Consdorf	natural	E
131212-89	*Cx. pipiens f. pipiens*	f	12.12.2013	Trou Hélène, Consdorf	nautral	T
130817-03	*Cx. pipiens f. pipiens*	f	17.08.2013	Minière Lalangebierg, Schifflange	artificial	T
131205-04	*Cx. pipiens* f. pipiens	f	05.12.2013	Cellar, Echternach	artificial	-
131205-05	*Cx. pipiens f. pipiens*	f	05.12.2013	Cellar, Echternach	artificial	-
131205-15	*Cx. pipiens f. pipiens*	f	05.12.2013	Cellar, Echternach	artificial	-
131212-04	*Cx. pipiens f. pipiens*	f	12.12.2013	Cave Chateau, Schoenfels	artificial	T
131212-40	*Cx. pipiens* f. pipiens	f	12.12.2013	Cave Chateau, Schoenfels	artificial	T
131212-43	*Cx. pipiens f. pipiens*	f	12.12.2013	Cave Chateau, Schoenfels	artificial	D
131212-44	*Cx. pipiens f. pipiens*	f	12.12.2013	Cave Chateau, Schoenfels	artificial	D
131214-12	*Cx. pipiens f. pipiens*	f	14.12.2013	Ardoisière, Merkholz	artificial	
140308-116	*Cx. pipiens f. pipiens*	f	08.03.2014	Eisebunns tunnel, Fouhren	artificial	T
140308-118	*Cx. pipiens f. pipiens*	f	08.03.2014	Eisebunns tunnel, Fouhren	artificial	T
150321-46	*Cx. pipiens f. pipiens*	f	21.03.2015	Eisebunns tunnel, Fouhren	artificial	T
150321-52	*Cx. pipiens f. pipiens*	f	21.03.2015	Eisebunns tunnel, Fouhren	artificial	T
150321-53	*Cx. pipiens f. pipiens*	f	21.03.2015	Eisebunns tunnel, Fouhren	artificial	D
140330-50	*Cx. pipiens f. pipiens*	f	30.03.2014	Tunnel bei Doihl, Rodange	artificial	T
140330-73	*Cx. pipiens f. pipiens*	f	30.03.2014	Tunnel bei Doihl, Rodange	artificial	T
150404-06	*Cx. pipiens f. pipiens*	f	04.04.2015	Hell, Berdorf	natural	T
150404-28	*Cx. torrentium*	f	04.04.2015	Hell, Berdorf	natural	T
150404-38	*Cx. pipiens f. pipiens*	f	04.04.2015	Hell, Berdorf	natural	D
150404-40	*Cx. pipiens f. pipiens*	f	04.04.2015	Hell, Berdorf	natural	D
131112-40	*Cx. pipiens f. pipiens*	f	12.11.2013	Galerie Harelerbaach, Harlange	artifical	T

131112-45	*Cx. pipiens* f. *pipiens*	f	12.11.2013	Galerie Harelerbaach, Harlange	artificial	T
131112-52	*Cx. torrentium*	f	12.11.2013	Galerie Harelerbaach, Harlange	artificial	T
