# Supplementary material for: Mosquitoes (Diptera: Culicidae) in the Dark—Highlighting the Importance of Genetically Identifying Mosquito Populations in Subterranean Environments of Central Europe
Source: Pathogens. 2021 Aug 26;10(9):1090. doi: 10.3390/pathogens10091090 (PMC8467396; doi:10.3390/pathogens10091090)
Supplement: Supplementary file 1 [file pathogens-10-01090-s001.zip › Table_S2.pdf]

Table S2; Mosquitoes sampled in subterranean sites in Germany (T=transition zone, D=dark zone)

ID	taxon	gender	sampling date	sampling site	artificial / natural/touristic	cave region
**SubCul001**	*Cx. pipiens* f. *pipiens*	f	07.04.2018	Swabian Jura, Gussmannhöhle	natural, touristic	D
**SubCul002**	*Cx. torrentium*	f	07.04.2018	Swabian Jura, Gussmannhöhle	natural, touristic	D
**SubCul003**	*Cx. pipiens* f. *pipiens*	f	07.04.2018	Swabian Jura, Gussmannhöhle	natural, touristic	D
**SubCul004**	*Cx. torrentium*	f	07.04.2018	Swabian Jura, Gussmannhöhle	natural, touristic	D
**SubCul006**	*Cx. pipiens* f. *pipiens*	f	31.10.2017 - 07.04.2018	Swabian Jura, Krebssteinhöhle	natural	D
**SubCul007**	*Cx. pipiens* f. *pipiens*	f	28.04.2018	Süntel Mts., Schillathöhle	natural, touristic	T
**SubCul008**	*Cx. pipiens* f. *pipiens*	f	28.04.2018	Süntel Mts., Schillathöhle	natural, touristic	T
**SubCul009**	*Cx. pipiens* f. *pipiens*	f	28.04.2018	Süntel Mts., Schillathöhle	natural, touristic	T
**SubCul010**	*Cx. pipiens* f. *pipiens*	f	28.04.2018	Süntel Mts., Schillathöhle	natural, touristic	T
**SubCul011**	Cx. *pipiens* f. *pipiens*	f	07.04.2018	Swabian Jura, Gussmannhöhle	natural, touristic	T
**SubCul012**	*Cx. torrentium*	f	07.04.2018	Swabian Jura, Gussmannhöhle	natural, touristic	T
**SubCul013**	*Cx. torrentium*	f	07.04.2018	Swabian Jura, Gussmannhöhle	natural, touristic	T
**SubCul014**	*Cx. pipiens* f. *pipiens*	f	07.04.2018	Swabian Jura, Gussmannhöhle	natural, touristic	T
**SubCul015**	*Cx. pipiens* f. *pipiens*	f	07.04.2018	Swabian Jura, Gussmannhöhle	natural, touristic	T
**SubCul016**	*Cx. torrentium*	f	07.04.2018	Swabian Jura, Gussmannhöhle	natural, touristic	T
**SubCul017**	*Cx. torrentium*	f	07.04.2018	Swabian Jura, Gussmannhöhle	natural, touristic	T
**SubCul018**	*Cx. pipiens* f. *pipiens*	f	07.04.2018	Swabian Jura, Wolfshöhle	natural	D
**SubCul019**	*Cx. torrentium*	f	07.04.2018	Swabian Jura, Wolfshöhle	natural	D
**SubCul020**	*Cx. pipiens* f. *pipiens*	f	07.04.2018	Swabian Jura, Wolfshöhle	natural	D
**SubCul021**	*Cx. pipiens* f. *pipiens*	f	07.04.2018	Swabian Jura, Wolfshöhle	natural	D
**SubCul022**	*Cx. pipiens* f. *pipiens*	f	07.04.2018	Swabian Jura, Wolfshöhle	natural	D

**SubCul028**	*Cx. pipiens* f. *pipiens*	f	07.04.2018	Swabian Jura, Krebssteinhöhle	natural	D
**SubCul029**	*Cx. pipiens* f. *pipiens*	f	07.04.2018	Swabian Jura, Krebssteinhöhle	natural	D
**SubCul030**	*Cx. pipiens* f. *pipiens*	f	07.04.2018	Swabian Jura, Krebssteinhöhle	natural	D
**SubCul031**	*Cx. pipiens* f. *pipiens*	f	07.04.2018	Swabian Jura, Gutenberger Höhle	natural, touristic	T
**SubCul032**	*Cx. pipiens* f. *pipiens*	f	07.04.2018	Swabian Jura, Gutenberger Höhle	natural, touristic	T
**SubCul033**	*Cx. pipiens* f. *pipiens*	f	07.04.2018	Swabian Jura, Gutenberger Höhle	natural, touristic	T
**SubCul034**	*Cx. pipiens* f. *pipiens*	f	07.04.2018	Swabian Jura, Gutenberger Höhle	natural, touristic	T
**SubCul035**	*Cx. pipiens* f. *pipiens* x *mol*	f	07.04.2018	Swabian Jura, Gutenberger Höhle	natural, touristic	T
**SubCul036**	*Cx. pipiens* f. *pipiens*	f	07.04.2018	Swabian Jura, Gutenberger Höhle	natural, touristic	T
**SubCul037**	*Cx. pipiens* f. *pipiens*	f	07.04.2018	Swabian Jura, Krebssteinhöhle	natural	T
**SubCul038**	*Cx. pipiens* f. *pipiens*	f	07.04.2018	Swabian Jura, Krebssteinhöhle	natural	T
**SubCul039**	*Cx. pipiens* f. *pipiens*	f	07.04.2018	Swabian Jura, Krebssteinhöhle	natural	T
**SubCul040**	*Cx. torrentium*	f	07.04.2018	Swabian Jura, Krebssteinhöhle	natural	T
**SubCul041**	*Cx. torrentium*	f	07.04.2018	Swabian Jura, Krebssteinhöhle	natural	T
**SubCul042**	*Cx. pipiens* f. *pipiens*	f	07.04.2018	Swabian Jura, Krebssteinhöhle	natural	T
**SubCul043**	*Cx. pipiens* f. *pipiens*	f	28.04.2018	Süntel Mts., Riesenberghöhle	natural	D
**SubCul044**	*Cx.* pipiens f. *pipiens*	f	28.04.2018	Süntel Mts., Riesenberghöhle	natural	D
**SubCul046**	*Cx. pipiens* f. *pipiens*	f	19.08.2017	Süntel Mts., Riesenberghöhle	natural	T
**SubCul048**	*Cx. pipiens* f. *pipiens*	f	19.08.2017	Süntel Mts., Riesenberghöhle	natural	T
**SubCul049**	*Cx. torrentium*	f	19.08.2017	Süntel Mts., Riesenberghöhle	natural	T
**SubCul050**	*Cx. torrentium*	f	12.09.2017	Harz Mts., Einhornhöhle	natural, touristic	D
**SubCul051**	*Cx. pipiens* f. *pipiens*	f	12.09.2017	Harz Mts., Einhornhöhle	natural, touristic	D

**SubCul052**	*Cx. pipiens* f. *pipiens*	f	12.09.2017	Harz Mts., Einhornhöhle	natural, touristic	D
**SubCul053**	*Cx. pipiens* f. *pipiens*	f	12.09.2017	Harz Mts., Einhornhöhle	natural, touristic	D
**SubCul055**	*Cx. pipiens* f. *pipiens*	f	30.07.2018	Swabian Jura, Krebssteinhöhle	natural	T
**SubCul057**	*Cx. pipiens* f. *pipiens*	f	12.09.2017	Harz Mts., Einhornhöhle	natural, touristic	T
**SubCul059**	*Cx. torrentium*	f	30.07.2018	Swabian Jura, Wolfshöhle	natural	D
**SubCul060**	*Cx. torrentium*	f	20.09.2017	Westerwald, Adventhöhle	natural	D
**SubCul061**	*Cx. pipiens* f. pipiens	f	19.08.2017	Süntel Mts., Schillathöhle	natural, touristic	T
**SubCul063**	*Cx. pipiens* f. pipiens	f	19.08.2017	Süntel Mts., Schillathöhle	natural, touristic	T
**SubCul064**	*Cx. pipiens* f. *pipiens*	f	19.08.2017	Süntel Mts., Schillathöhle	natural, touristic	T
**SubCul065**	*Cx. torrentium*	f	20.09.2017	Westerwald, Adventhöhle	natural	T
**SubCul066**	*Cx. pipiens* f. *mol*	f	20.09.2017	Westerwald, Adventhöhle	natural	T
**SubCul067**	*Cx. torrentium*	f	12.09.2017	Harz Mts., Weingartenloch	natural	D
**SubCul068**	*Cx. pipiens* f. *pipiens*	f	12.09.2017	Harz Mts., Weingartenloch	natural	D
**SubCul069**	*Cx. torrentium*	f	12.09.2017	Harz Mts., Weingartenloch	natural	D
**SubCul070**	*Cx. torrentium*	f	12.09.2017	Harz Mts., Weingartenloch	natural	D
**SubCul072**	*Cx. pipiens* f. *pipiens*	f	12.09.2017	Harz Mts., Weingartenloch	natural	D
**SubCul073**	*Cx. torrentium*	f	12.09.2017	Harz Mts., Weingartenloch	natural	D
**SubCul074**	*Cx. torrentium*	f	12.09.2017	Harz Mts., Weingartenloch	natural	D
**SubCul076**	Cx. *torrentium*	f	12.09.2017	Harz Mts., Weingartenloch	natural	D
**SubCul077**	Cx. *torrentium*	f	12.09.2017	Harz Mts., Weingartenloch	natural	D
**SubCul078**	*Cx. torrentium*	f	12.09.2017	Harz Mts., Weingartenloch	natural	D
**SubCul080**	*Cx. pipiens* f. *pipiens*	f	12.09.2017	Harz Mts., Weingartenloch	natural	D
**SubCul081**	*Cx. torrentium*	f	12.09.2017	Harz Mts., Weingartenloch	natural	D
**SubCul091**	*Cx. pipiens* f. *pipiens*	f	30.07.2018	Swabian Jura, Gussmannhöhle	natural, touristic	T
**SubCul092**	*Cx. pipiens* f. *pipiens*	f	09.10.2017	Franconian Switzerland, Mammuthöhle	natural	D

**SubCul093**	*Cx. pipiens* f. *pipiens*	f	09.10.2017	Franconian Switzerland, Mammuthöhle	natural	D
**SubCul094**	*Cx. pipiens* f. *pipiens*	f	09.10.2017	Franconian Switzerland, Mammuthöhle	natural	D
**SubCul095**	Cx. *pipiens* f. *pipiens*	f	14.10.2017	Swabian Jura, Gussmannhöhle	natural, touristic	D
**SubCul096**	*Cx. torrentium*	f	14.10.2017	Swabian Jura, Gussmannhöhle	natural, touristic	D
**SubCul097**	*Cx. pipiens* f. *pipiens*	f	14.10.2017	Swabian Jura, Gussmannhöhle	natural, touristic	D
**SubCul098**	*Cx. pipiens* f. *pipiens*	f	14.10.2017	Swabian Jura, Gussmannhöhle	natural, touristic	D
**SubCul100**	*Cx.* pipiens f. *pipiens*	f	14.10.2017	Swabian Jura, Gussmannhöhle	natural, touristic	D
**SubCul101**	*Cx. pipiens* f. *pipiens*	f	30.07.2018	Swabian Jura, Krebssteinhöhle	natural	D
**SubCul102**	*Cx. pipiens* f. *pipiens*	f	14.10.2017	Swabian Jura, Gussmannhöhle	natural, touristic	T
**SubCul103**	*Cx. pipiens* f. *pipiens*	f	14.10.2017	Swabian Jura, Gussmannhöhle	natural, touristic	T
**SubCul104**	*Cx. pipiens* f. *pipiens*	f	14.10.2017	Swabian Jura, Gussmannhöhle	natural, touristic	T
**SubCul105**	*Cx. pipiens* f. *pipiens*	f	14.10.2017	Swabian Jura, Gussmannhöhle	natural, touristic	T
**SubCul106**	*Cx. torrentium*	f	14.10.2017	Swabian Jura, Gussmannhöhle	natural, touristic	T
**SubCul107**	*Cx.* pipiens f. *pipiens*	f	14.10.2017	Swabian Jura, Gussmannhöhle	natural, touristic	T
**SubCul108**	*Cx. torrentium*	f	14.10.2017	Swabian Jura, Gussmannhöhle	natural, touristic	T
**SubCul109**	*Cx. pipiens* f. *pipiens*	f	14.10.2017	Swabian Jura, Gussmannhöhle	natural, touristic	T
**SubCul110**	*Cx. pipiens* f. *pipiens*	f	14.10.2017	Swabian Jura, Gussmannhöhle	natural, touristic	T
**SubCul111**	*Cx. pipiens* f. *pipiens*	f	14.10.2017	Swabian Jura, Gussmannhöhle	natural, touristic	T
**SubCul112**	*Cx. pipiens* f. *pipiens*	f	09.10.2017	Franconian Switzerland, Mammuthöhle	natural	T
**SubCul114**	*Cx. torrentium*	f	09.10.2017	Franconian Switzerland, Mammuthöhle	natural	T
**SubCul115**	*Cx. pipiens* f. *pipiens*	f	14.10.2017	Swabian Jura, Wolfshöhle	natural	D
**SubCul116**	*Cx. torrentium*	f	14.10.2017	Swabian Jura, Wolfshöhle	natural	D

**SubCul117**	*Cx. pipiens* f. *pipiens*	f	14.10.2017	Swabian Jura, Wolfshöhle	natural	D
**SubCul118**	Cx. *pipiens* f. *pipiens*	f	14.10.2017	Swabian Jura, Wolfshöhle	natural	D
**SubCul119**	*Cx. pipiens* f. *pipiens*	f	14.10.2017	Swabian Jura, Wolfshöhle	natural	D
**SubCul120**	*Cx. torrentium*	f	14.10.2017	Swabian Jura, Wolfshöhle	natural	D
**SubCul121**	*Cx. pipiens* f. *pipiens*	f	14.10.2017	Swabian Jura, Wolfshöhle	natural	T
**SubCul122**	*Cx. torrentium*	f	14.10.2017	Swabian Jura, Wolfshöhle	natural	T
**SubCul123**	*Cx. torrentium*	f	14.10.2017	Swabian Jura, Wolfshöhle	natural	T
**SubCul124**	*Cx. torrentium*	f	14.10.2017	Swabian Jura, Wolfshöhle	natural	T
**SubCul125**	*Cx. pipiens* f. *pipiens*	f	14.10.2017	Swabian Jura, Wolfshöhle	natural	T
**SubCul127**	*Cx. pipiens* f. *pipiens*	f	14.10.2017	Swabian Jura, Krebssteinhöhle	natural	T
**SubCul128**	*Cx. pipiens* f. *pipiens*	f	14.10.2017	Swabian Jura, Krebssteinhöhle	natural	T
**SubCul129**	*Cx. pipiens* f. *pipiens*	f	14.10.2017	Swabian Jura, Krebssteinhöhle	natural	T
**SubCul130**	*Cx. torrentium*	f	14.10.2017	Swabian Jura, Krebssteinhöhle	natural	T
**SubCul131**	*Cx. pipiens* f. *pipiens*	f	14.10.2017	Swabian Jura, Krebssteinhöhle	natural	T
**SubCul133**	*Cx. pipiens* f. *pipiens*	f	14.10.2017	Swabian Jura, Krebssteinhöhle	natural	T
**SubCul134**	*Cx. pipiens* f. *pipiens*	f	14.10.2017	Swabian Jura, Krebssteinhöhle	natural	T
**SubCul137**	*Cx. pipiens* f. *pipiens*	f	14.10.2017	Swabian Jura, Gutenberghöhle	natural, touristic	D
**SubCul139**	*Cx. pipiens* f. *pipiens*	f	14.10.2017	Swabian Jura, Gutenberghöhle	natural, touristic	D
**SubCul140**	*Cx. pipiens* f. *pipiens*	f	14.10.2017	Swabian Jura, Gutenberghöhle	natural, touristic	D
**SubCul141**	*Cx. torrentium*	f	14.10.2017	Swabian Jura, Gutenberghöhle	natural, touristic	D
**SubCul144**	*Cx.* pipiens f. *pipiens* X *molestus*	f	14.10.2017	Swabian Jura, Gutenberghöhle	natural, touristic	D
**SubCul145**	*Cx. pipiens* f. *pipiens*	f	14.10.2017	Swabian Jura, Gutenberghöhle	natural, touristic	D
**SubCul146**	*Cx. torrentium*	f	14.10.2017	Swabian Jura, Gutenberghöhle	natural, touristic	D

**SubCul147**	*Cx. pipiens* f. *pipiens*	m	14.10.2017	Swabian Jura, Gutenberghöhle	natural, touristic	T
**SubCul149**	*Cx. pipiens* f. molestus	f	14.10.2017	Swabian Jura, Gutenberghöhle	natural, touristic	T
**SubCul150**	*Cx. pipiens* f. *pipiens*	f	14.10.2017	Swabian Jura, Gutenberghöhle	natural, touristic	T
**SubCul151**	*Cx. pipiens* f. *pipiens*	f	14.10.2017	Swabian Jura, Gutenberghöhle	natural, touristic	T
**SubCul152**	*Cx. pipiens* f. *pipiens* X *molestus*	f	14.10.2017	Swabian Jura, Gutenberghöhle	natural, touristic	T
**SubCul153**	*Cx. pipiens* f. *pipiens*	f	14.10.2017	Swabian Jura, Gutenberghöhle	natural, touristic	T
**SubCul154**	*Cx. pipiens* f. *pipiens*	f	14.10.2017	Swabian Jura, Gutenberghöhle	natural, touristic	T
**SubCul155**	*Cx. pipiens* f. *pipiens*	f	14.10.2017	Swabian Jura, Gutenberghöhle	natural, touristic	T
**SubCul156**	*Cx. pipiens* f. *pipiens*	f	14.10.2017	Swabian Jura, Gutenberghöhle	natural, touristic	T
**SubCul157**	*Cx. pipiens* f. *pipiens* X *molestus*	f	14.10.2017	Swabian Jura, Gutenberghöhle	natural, touristic	T
**SubCul158**	*Cx. torrentium*	f	07.10.2017	Harz Mts., Weingartenloch	natural	T
**SubCul159**	*Cx. pipiens* f. *pipiens*	f	07.10.2017	Harz Mts., Weingartenloch	natural	T
**SubCul160**	*Cx. pipiens* f. *pipiens*	f	07.10.2017	Harz Mts., Weingartenloch	natural	T
**SubCul161**	*Cx. pipiens* f. *pipiens*	f	07.10.2017	Harz Mts., Weingartenloch	natural	T
**SubCul162**	*Cx. pipiens* f. *pipiens*	f	07.10.2017	Harz Mts., Weingartenloch	natural	T
**SubCul163**	*Cx. torrentium*	f	07.10.2017	Harz Mts., Weingartenloch	natural	T
**SubCul165**	*Cx. pipiens* f. *pipiens*	f	02.05.2018	Franconian Switzerland, Mammuthöhle	natural	D
**SubCul168**	*Cx. torrentium*	f	06.04.2018	Westerwald, Adventhöhle	natural	T
**SubCul169**	*Cx. torrentium*	f	06.04.2018	Westerwald, Adventhöhle	natural	T
**SubCul170**	*Cx. torrentium*	f	07.04.2018	Swabian Jura, Wolfshöhle	natural	T
**SubCul171**	*Cx. torrentium*	f	07.04.2018	Swabian Jura, Wolfshöhle	natural	T
**SubCul172**	Cx. *pipiens* f. *pipiens*	f	07.04.2018	Swabian Jura, Wolfshöhle	natural	T

**SubCul173**	*Cx. pipiens* f. *pipiens*	f	07.04.2018	Swabian Jura, Wolfshöhle	natural	T
**SubCul174**	*Cx. pipiens* f. *pipiens*	f	07.04.2018	Swabian Jura, Wolfshöhle	natural	T
**SubCul175**	*Cx. torrentium*	f	07.04.2018	Swabian Jura, Wolfshöhle	natural	T
**SubCul176**	*Cx. torrentium*	f	07.04.2018	Swabian Jura, Wolfshöhle	natural	T
**SubCul177**	*Cx. torrentium*	f	07.04.2018	Swabian Jura, Wolfshöhle	natural	T
**SubCul178**	*Cx. pipiens* f. *pipiens*	f	07.04.2018	Swabian Jura, Wolfshöhle	natural	T
**SubCul179**	*Cx. pipiens* f. *pipiens*	f	20.04.2018	Harz Mts., Weingartenloch	natural	D
**SubCul180**	*Cx. pipiens* f. *pipiens*	f	28.04.2018	Süntel Mts., Riesenberghöhle	natural	T
**SubCul181**	*Cx. pipiens* f. *pipiens*	f	28.04.2018	Süntel Mts., Riesenberghöhle	natural	T
**SubCul182**	*Cx. pipiens* f. pipiens	f	28.04.2018	Süntel Mts., Riesenberghöhle	natural	T
**SubCul183**	*Cx. pipiens* f. *pipiens*	f	28.04.2018	Süntel Mts., Riesenberghöhle	natural	T
**SubCul184**	*Cx. pipiens* f. *pipiens*	f	28.04.2018	Süntel Mts., Riesenberghöhle	natural	T
**SubCul186**	*Cx. pipiens* f. *pipiens*	f	28.04.2018	Süntel Mts., Riesenberghöhle	natural	T
**SubCul187**	*Cx. pipiens* f. *pipiens*	f	28.04.2018	Süntel Mts., Riesenberghöhle	natural	T
**SubCul188**	*Cx. pipiens* f. *pipiens*	f	31.10.2017 - 07.04.2018	Swabian Jura, Wolfshöhle	natural	D
**SubCul189**	*Cx. pipiens* f. *pipiens*	m	30.06.2018	Süntel Mts., Schillathöhle	natural, touristic	T
